# Ligand activated progesterone receptor B drives autophagy-senescence transition through a Beclin-1/Bcl-2 dependent mechanism in human breast cancer cells

**DOI:** 10.18632/oncotarget.10799

**Published:** 2016-07-23

**Authors:** Francesca De Amicis, Carmela Guido, Marta Santoro, Francesca Giordano, Ada Donà, Pietro Rizza, Michele Pellegrino, Ida Perrotta, Daniela Bonofiglio, Diego Sisci, Maria Luisa Panno, Donatella Tramontano, Saveria Aquila, Sebastiano Andò

**Affiliations:** ^1^ Centro Sanitario, University of Calabria, Rende, Italy; ^2^ Department of Pharmacy, Health Science and Nutrition, University of Calabria, Rende, Italy; ^3^ DiBEST University of Calabria, Rende, Italy; ^4^ Department of Molecular Medicine and Medical Biotechnologies, University of Naples “Federico II”, Naples, Italy

**Keywords:** progesterone, Bcl-2, cell cycle arrest, pRb, p16

## Abstract

Loss of progesterone-receptors (PR) expression is associated with breast cancer progression. Herein we provide evidence that OHPg/PR-B through Beclin-1 evoke autophagy-senescence transition, in breast cancer cells. Specifically, OHPg increases Beclin-1 expression through a transcriptional mechanism due to the occupancy of Beclin-1 promoter by PR-B, together with the transcriptional coactivator SRC-2. This complex binds at a canonical half progesterone responsive element, which is fundamental for OHPg effects, as shown by site-directed mutagenesis. Beside, OHPg *via* non-genomic action rapidly activates JNK, which phosphorylates Bcl-2, producing the functional release from Beclin-1 interaction. This is not linked to an efficient autophagic flux, since p62 levels, marker of degradation *via* lysosomes, were not reduced after sustained OHPg stimulus. Instead, the cell cycle inhibitor p27 was induced, together with an irreversible G1 arrest, hallmark of cellular senescence. Specifically the increase of senescence-associated β-galactosidase activity was blocked by Bcl-2 siRNA but also by Beclin-1 siRNA. Collectively these findings support the importance of PR-B expression in breast cancer cells, thus targeting PR-B may be a useful strategy to provide additional approaches to existing therapies for breast cancer patients.

## INTRODUCTION

Estrogen (ER) and Progesterone Receptors (PR) and their ligands, known factors involved in breast cancer initiation and progression, are able to determine a dynamic bio-molecular context correlated with breast tumor heterogeneity [1, 2]. This is the major problem which critically influences the response of patients to therapy [3].

Progesterone receptors action in mediating progesterone effects is highly context dependent and heavily influenced by post-translational modifications [4] as well as cofactor availability [5]. Several independent *in vitro* studies have demonstrated the inhibitory action of progesterone on breast cancer cell growth [6–8] and in agreement with these findings in a very recent paper we report that hydroxyprogesterone (OHPg), *via* PR-B isoform, leads to a reduced cell survival due to autophagy induction [9] through PTEN up-regulation.

Autophagy, is a genetically regulated mechanism responsible for the turnover of cellular proteins and damaged organelles which exhibits a complex control, crucially depending on mTOR, through the switch of several signal pathways, such as LKB1/AMPK, MEM/ERK and PI3K/Akt [10]. The activation of the process can be oncogenic by contributing to tumor cell survival [11], besides the data from autophagy defective Beclin-1 knockout mice also suggest a tumor suppressive activity [12, 13].

Beclin-1, the master regulator, interacts with PI3KIII during autophagosomal membrane engulfing of damaged cytoplasmic organelles and long-lived proteins [14]. The function of Beclin-1 is, in part, defined by its interaction with the anti-apoptotic gene products, Bcl-2 and Bcl-xL [15]. The dissociation of Beclin-1 from Bcl-2 is critical for autophagy induction [16] and is regulated by many proteins and signal pathways. Specifically JNK1 or ERK1/2-mediated phosphorylation of Bcl-2 or DAPK-mediated phosphorylation of Beclin-1 [17–18].

Recent acquisitions suggest that autophagy may serve as a switch to shift the cell fate to senescence, providing a protective mechanism against the tumorigenic factors [13, 19–21]. Progestins activate nuclear PR-B leading to robust induction of cell cycle mediators of senescence in ovarian cancer cells [22].

Herein, in the aim of further explore OHPg/PR-B protective effects in breast cancer we investigate how PR-B prolonged activation, driving autophagy through Beclin-1, may influence cell cycle control by intercepting a signalling pathway responsible for autophagy-senescence transition.

## RESULTS

### OHPg increases Beclin-1 through a genomic mechanism

**Figure 1:**
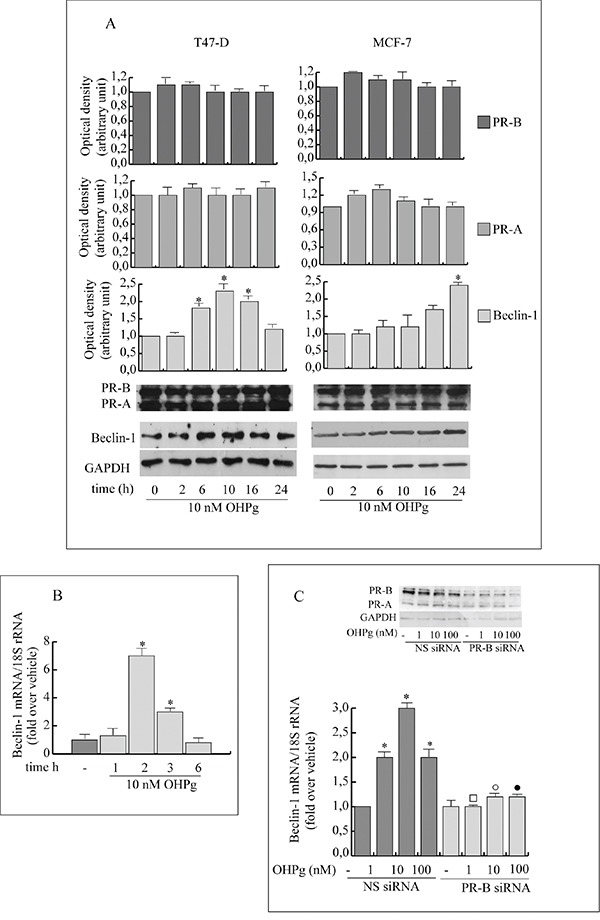
OHPg through PR-B up-regulates Beclin-1 protein and mRNA levels in breast cancer cells **A.** Western blotting analysis of Beclin-1, PR-A and PR-B. T47-D and MCF-7 breast cancer cells treated with 10 nM OHPg at different times as indicated. GAPDH was used as loading control. Autoradiograph shows the results of one representative experiment out of three. **B.** Real-time PCR assay of Beclin-1 mRNA expression in T47-D cells treated at different times as indicated. **C.** Real-time PCR assay of Beclin-1 mRNA expression in MCF-7 cells transfected with non specific (NS)- or targeted against PR-B siRNA treated with vehicle (−) or OHPg for 24h as indicated. 18S rRNA was determined as control. Columns are the mean of three independent experiments each in triplicate; bars, SD; * p ≤0.05 vs vehicle treated cells; ▪ p ≤0.05 vs. 1 nM OHPg; ○ p ≤0.05 vs. 10 nM OHPg ● p ≤0.05 vs 100 nM OHPg. In the insert, Western blotting analysis of PR-B expression in MCF-7 cells in the same experimental conditions. GAPDH was used as loading control.

**Figure 2:**
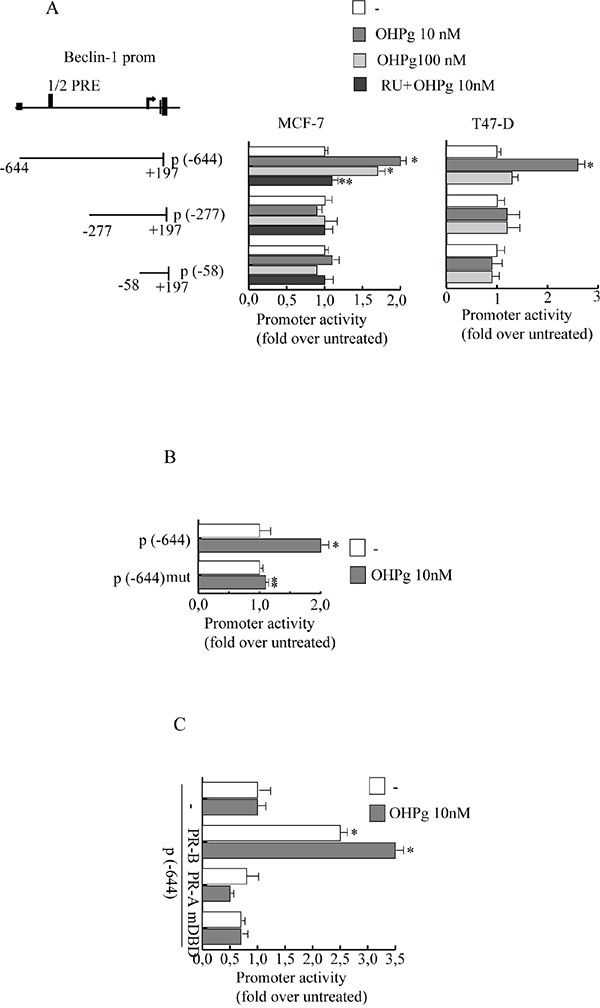
OHPg transactivates Beclin-1 promoter gene in breast cancer cells **A.** left panel. Schematic representation of deletion fragments of the Beclin-1 gene promoter. Right panel. Constructs depicted were transiently transfected in MCF-7 and T47-D cells, treated with vehicle (−) or OHPg or 1μM RU 486 +10 nM OHPg as indicated. Columns are mean of three independent experiments and expressed as fold change over untreated, which was assumed to be 1; bars SD; * p ≤0.05 vs vehicle. ** p ≤0.05 vs OHPg 10 nM **B.** Site-directed mutagenesis of the 1/2PRE site. p(−644) and p(−644) mut promoter constructs were transfected into MCF-7 cells, and activity was assessed in the absence or presence of 10 nM OHPg. After 24 h, cells were harvested and luciferase activities were determined; bars SD; * p ≤0.05 vs vehicle. ** p ≤0.05 vs p(−644) 10 nM OHPg. **C.** MDA-MB231 cells were co-transfected with the Beclin-1 promoter construct p(−644) and PR-B or PR-A or mDBD expression vector, and then treated for 24 h with vehicle (−) or 10 nM OHPg; bars, SD; * p ≤0.05 vs vehicle.

Beclin-1 is a component of the class IIIPI3 kinase complex which is required for autophagy activation by various stimuli [23, 24]. Stemming from our data indicating that 10 nM OHPg *via* PR-B [9] increases protein and mRNA levels of Beclin-1 in T47-D and MCF-7 cells (Figure 1A-1C), we next investigated the effects of OHPg/PR-B on Beclin-1 gene transcription. To this aim we performed transient transfection studies by using the 5′ flanking region of Beclin-1 expression vector and two different deleted constructs (Figure 2A, *left panel*) [23]. 10 nM OHPg significantly increased the activity of the construct p(−644) in MCF-7 cells, as depicted in Figure 2A (*right panel)* and co-treatment with RU-486 (RU), a synthetic steroid with anti-progesterone receptor activity [25], counteracted this effect. None noticeable effect was observed in MCF-7 as well as in T47-D cells upon OHPg in the presence of p(−277) and p(−58) constructs, therefore the region between −644 bp to −277 bp holds important regulatory elements implicated in OHPg-mediated up-regulation of Beclin-1 promoter activity.

For instance, sequence analysis identified a canonical half progesterone responsive element (1/2PRE) located from −512 bp to −507 bp. To investigate the potential role of this specific site we performed site-directed mutagenesis and we changed 3 bp of the 1/2PRE to ensure that the altered binding site would not be recognizable by the PR. Transient transfections were performed in MCF-7 cells using multiple independent clones containing the desiderated mutation. We found that the activity of construct mut(−644) carrying the mutation in the 1/2PRE site, was unaffected by OHPg stimulus (Figure 2B). These results indicate that the 1/2PRE element is required for induction of Beclin-1 promoter activity by OHPg.

To prove PR-B specific involvement we used PR-negative MDA-MB-231 co-transfected with expression plasmids encoding either PR-B, PR-A or PR mutated in the DNA binding domain (mDBD) (Figure 2C). PR-B isoform expression itself was able to significantly increase the activity of Beclin-1 promoter which was further enhanced by OHPg treatment. Instead, ectopic expression of PR-A isoform or mDBD did not exert significant modulatory effects. Altogether, these data strongly indicate that PR-B *via* 1/2PRE represents a fundamental activator of Beclin-1 transcription by OHPg.

**Figure 3:**
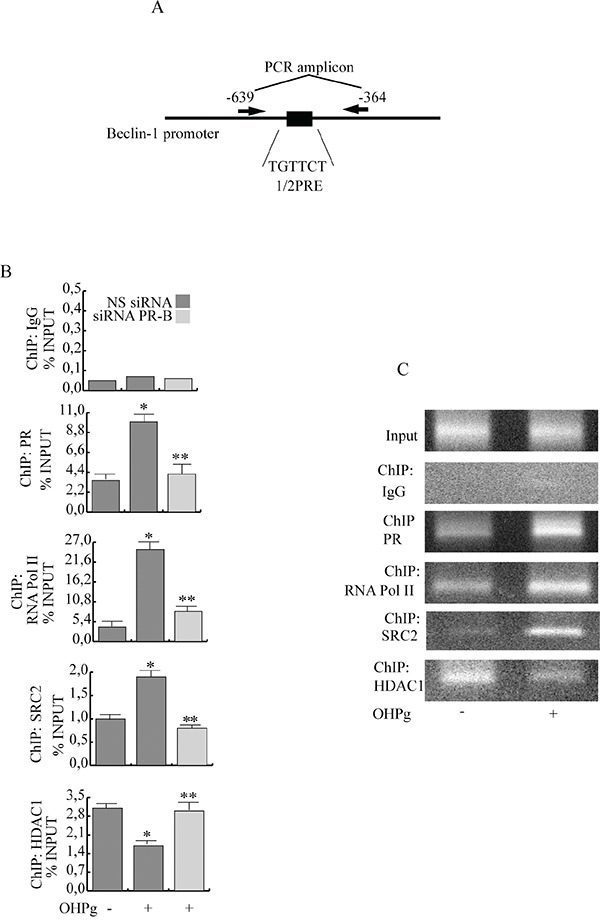
Ligand-activated PR-B binds the Beclin-1 gene promoter together with SRC-2 coactivator **A.** Schematic representation of Beclin-1 promoter region containing the 1/2PRE site. **B.** ChIP-qPCR. MCF-7 cells transfected with non specific (NS) or targeted against PR-B siRNA treated with vehicle (−) or 10 nM OHPg. ChIP-qPCR was performed using primers as depicted in (A) and antibodies are used as indicated. IgG was used as control. Columns are the mean of three independent experiments. bars, SD; * p ≤0.05 vs vehicle. ** p ≤0.05 vs 10 nM OHPg. **C.** ChIP assay. T47-D treated with vehicle (−) or 10 nM OHPg. DNA Input, loading control. PCR was performed using primers as depicted in (A) antibodies are used as indicated. IgG was used as control. Images are representative of three independent experiments.

To investigate PR-B recruitment to the Beclin-1 gene promoter, we performed ChIP assays. MCF-7 cells were exposed for 6h to either control vehicle or 10 nM OHPg, after which chromatin was cross-linked with formaldehyde, and protein-DNA complexes were immunoprecipitated with antibodies directed against PR. The qPCR primers used encompass the 1/2PRE site we identified within the Beclin-1 promoter (Figure 3A). Results obtained after OHPg treatment demonstrate an enhanced recruitment of PR and RNA polymerase II to the Beclin-1 promoter and a reduction of HDAC1 indicating that the chromatin is in a more permissive environment for Beclin-1 gene transcription. Among the different PR coactivators previously described [26], some authors report that SRC-2 is critical for progesterone dependent function in breast morphogenesis [27]. We found that SRC-2 was present at the Beclin-1 promoter encompassing the half-PRE site, in a ligand-dependent manner, in contrast SRC-1 was absent (data not shown). All the above described effects were reversed by PR-B siRNA (Figure 3B). As a control, we did not see recruitment to an unrelated Beclin-1 promoter region located upstream of the 1/2PRE site (data not shown). Similar results were obtained in T47-D cells (Figure 3C).

**Figure 4:**
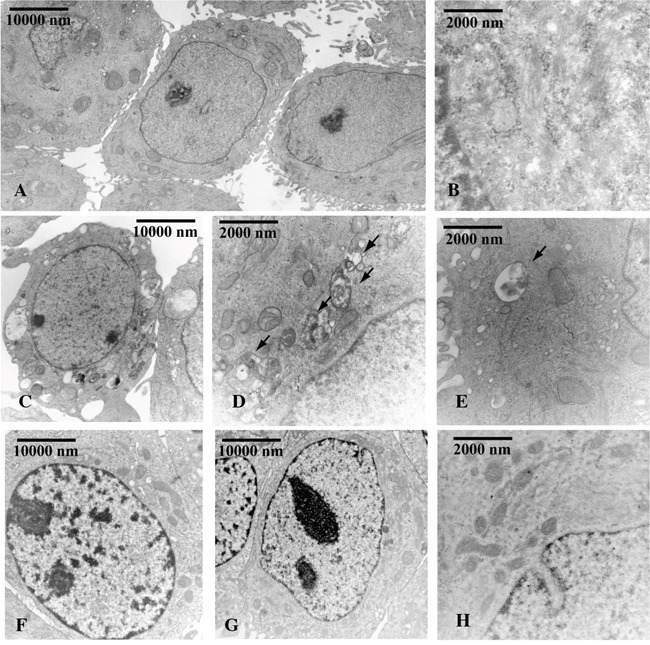
Beclin-1 is involved in OHPg induced autophagy in MCF-7 cells TEM analysis was conducted in MCF-7 cells transfected with NS siRNA **A-E.** or with Beclin-1 siRNA **F-H.** treated with vehicle (−) A, B. or 10 nM OHPg C-H. for 24 h. Black arrows D, E. indicate the autophagic bodies. Figure shows the results of one representative out of three independent experiments. Images magnification: a 6300X, b 31500X, c 6300X, d 25000X, e 31500X, f 10000X, g 10000X, h 31500X.

Next to demonstrate that Beclin-1 levels determined by OHPg stimulus, could mediate autophagy initiation [9], we performed siRNA studies. Accordingly with our previous data, OHPg treatment induced the accumulation of autophagic vacuoles in MCF-7 cells after 24h (Figure 4C, 4D, 4E) compared with vehicle treated cells (Figure 4A, 4B) as monitored by transmission electron microscopy (TEM) and the specific silencing of Beclin-1 greatly attenuated the effect produced by OHPg (Figure 4F, 4G, 4H).

### OHPg reduces Beclin-1/Bcl-2 interaction through a JNK-mediated rapid and sustained phosphorylation of Bcl-2

**Figure 5:**
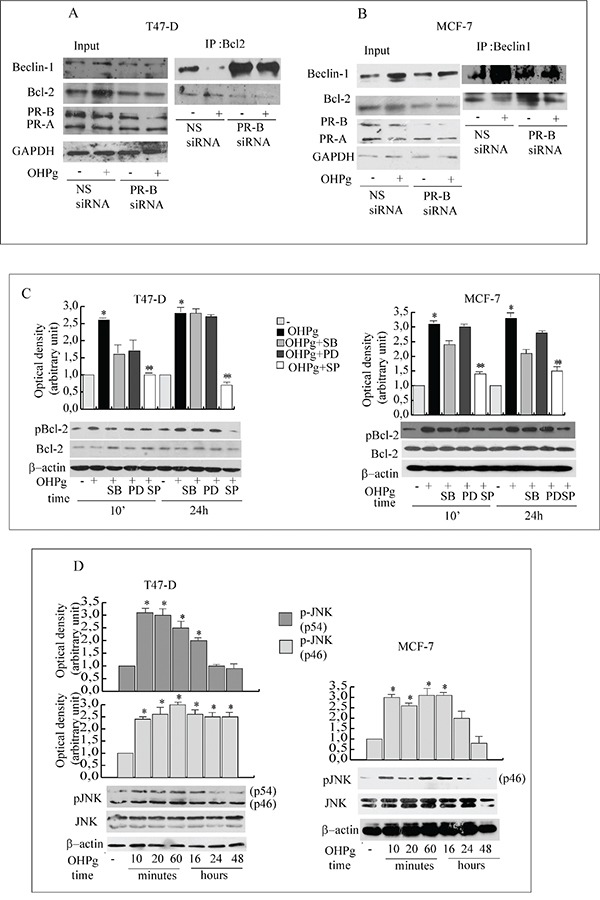
OHPg via JNK activation phosphorylates Bcl-2 and reduces Beclin-1/Bcl-2 interaction **A, B.** Co-immunoprecipitation analysis. Total extracts from T47-D (A) and MCF-7 cells (B) transfected with non specific (NS) or targeted against PR-B siRNA treated with vehicle (−) or 10 nM OHPg for 24h, were used for input, or for immunoprecipitation by using specific anti-Bcl-2 or anti-Beclin-1 antibody as indicated. Filters were blotted with anti-Bcl-2 or Beclin-1 or PR or GAPDH antibody **C.** Western blotting analysis of p-Bcl-2 and Bcl-2. T47-D and MCF-7 cells were treated with 10 nM OHPg, 10 μM SB 203580, 1 μM PD 98059, 20 μM SP 600125 as indicated. Β-actin was used as loading control. Autoradiographs show the results of one representative experiment out of three. Columns, are mean of three independent experiments in which band intensities were evaluated in terms of optical density arbitrary units and expressed as fold over vehicle, which was assumed to be 1; bars, SD. * p ≤0.05 vs vehicle treated cells. ** p ≤0.05 vs OHPg. **D.** Western blotting analysis of p-JNK/JNK. T47-D and MCF-7 cells were treated with 10 nM OHPg as indicated. Β-actin was used as loading control. Autoradiographs show the results of one representative experiment. * p ≤0.05 vs vehicle treated cells.

Beclin-1 specific function in autophagy is inhibited by its interaction with Bcl-2 which dissociates once phosphorylated [17, 18]. Thus we investigated the effect of OHPg/PR-B on Beclin-1/Bcl-2 complex formation in T47-D and MCF-7 cells (Figure 5A, 5B). As shown by our IP studies, Bcl-2 associated with Beclin-1 in untreated cells. Worthy of note, this association decreased upon 24h of OHPg stimulation and specific silencing of PR-B abrogated this effect. IgG controls were performed (data not shown). Beside we observed that Bcl-2 was rapidly and sustained iper-phosphorylated after OHPg exposure in both cell types (Figure 5C).

C-jun N-terminal protein kinase (JNK) and the MAPK family members phosphorylate Bcl-2 at multiple sites [17, 28] thus we first evaluated whether JNK could be activated by OHPg (Figure 5D). A sustained increase of p-JNK was detected starting from 10′ in treated cells although total levels of JNK were equivalent, while p-p38 and p-MAPKs were weakly altered by OHPg (Supplementary Figure 1). Accordingly, pretreatment with 10 μM SB203580 (p38 inhibitor) or 1 μM PD98059 (MAPKs inhibitor) did not modified pBcl-2 levels, instead pretreatment with 20 μM SP600125 (JNK inhibitor) greatly counteracted the Bcl-2 phopshorylation induced by 24h of OHPg stimulation (Figure 5C) supporting the l role of JNK activation in the latter event.

Autophagy may represent: a) multistep pathway along which primarily long-lived proteins are degraded or b) a death-induced mechanism [12, 13, 19].

**Figure 6:**
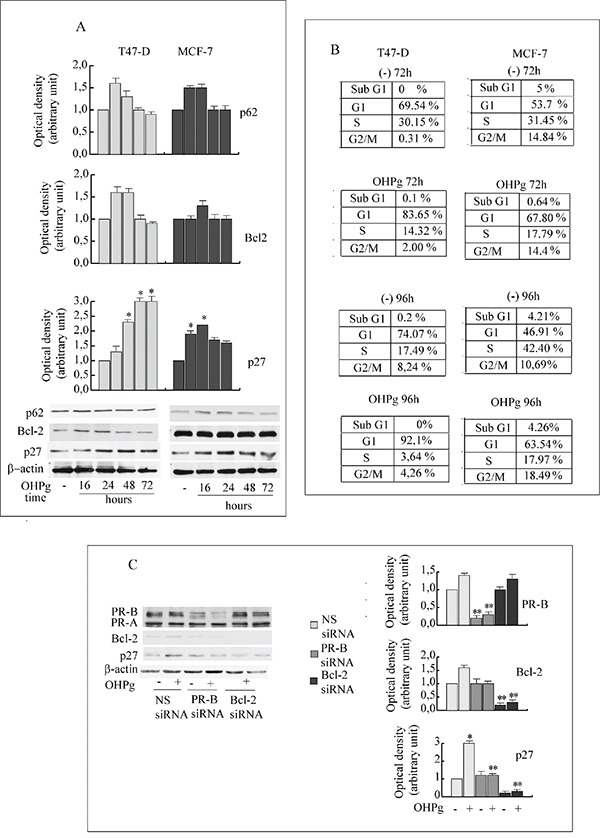
OHPg/PR-B induce irreversible G1 arrest in breast cancer cells **A.** Western blot analysis of p62, Bcl-2 and p27 expression in T47-D and MCF-7 cells treated with 10 nM OHPg as indicated. β-actin was used as loading control. Autoradiographs show the results of one representative experiment. Columns, are mean of three independent experiments in which band intensities were evaluated in terms of optical density arbitrary units and expressed as fold over vehicle, which was assumed to be 1; bars, SD * p ≤0.05 vs vehicle treated cells. **B.** FACS analysis. Cells were exposed to vehicle (−), 10 nM OHPgM as indicated. Data are representative of three independent experiments. **C.** Western blot analysis of PR-B, p27 and Bcl-2 expression in T47-D cells transfected with non specific (NS) or targeted against PR-B or Bcl-2 siRNA treated with vehicle (−) or 10 nM OHPg for 72h. Autoradiographs show the results of one representative experiment. Columns, are mean of three independent experiments in which band intensities were evaluated in terms of optical density arbitrary units and expressed as fold over vehicle, which was assumed to be 1; bars, SD * p ≤0.05 vs vehicle treated cells. ** p ≤0.05 vs NS siRNA.

TUNEL assay results indicated that prolonged OHPg stimulus did not produce evidence of apoptosis (data not shown). Surprisingly, in the same experimental conditions we observed the accumulation of p62, a marker of degradation via the lysosomes [29] in T47-D as well as in MCF-7 cells (Figure 6A), suggesting a deficient autophagic clearance of damaged proteins.

### OHPg/PR-B induce irreversible G1 arrest in breast cancer cells

Recent acquisitions sustain that the increase of impaired proteins may contribute to cellular senescence [30], described as an irreversible cell cycle arrest [31].

FACS analyses (Figure 6B) of T47-D cells show that after 72h of OHPg treatment an approximately 83% of cells accumulated in G1 phase as compared to ~69% of control, with a concomitant ~2-fold reduction of the population in the S-phase. The G1 arrest was persistent throughout 96h of OHPg treatment. To analyze whether the cell cycle block was reversible, OHPg was removed after 96h, by washing the cells and fresh medium was added for further 2 days. Percentage of T47-D cells in the G1/G0 phase of cell cycle remains unchanged (data not shown). Similar results were obtained in MCF-7 cells (Figure 6B).

Recent acquisitions suggest that cell cycle progression may be inhibited by Bcl-2 itself, *via* p27 KIP1 (p27) upregulation. [32, 33]. Accordingly, we observed an increase in the amount of p27 starting from 24h of OHPg treatment and sustained until 72h, in T47-D and MCF-7 cells (Figure 6A).

These effects were greatly counteracted in the presence of specific PR-B or Bcl-2 siRNA (Figure 6C) addressing the crucial role in the regulation of p27 levels.

### OHPg/PR-B via Bcl-2 drive the transition from autophagy to senescence

**Figure 7:**
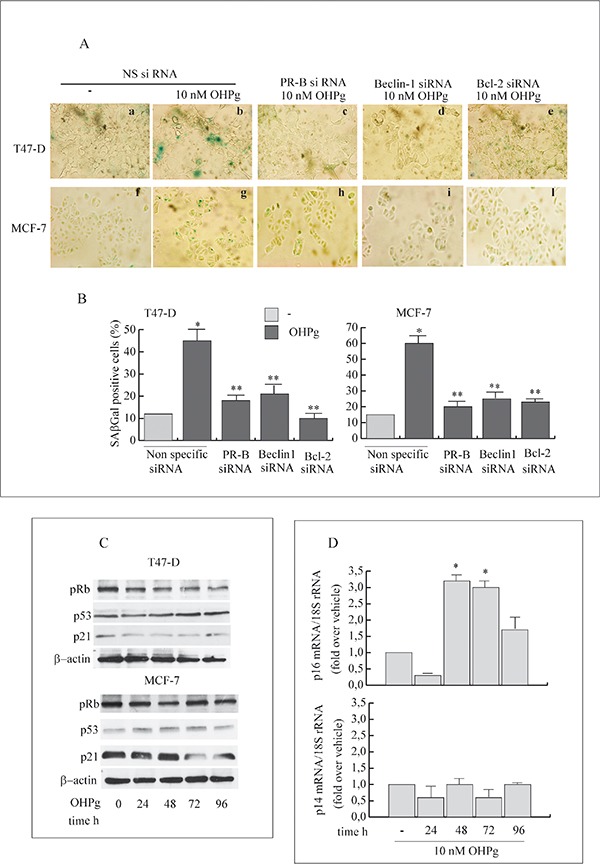
OHPg/PR-B induce cellular senescence and influence p16-pRb pathway in breast cancer cells **A.** Representative staining for SAβGal activity of T47-D (a-e) and MCF-7 cells (f-l) transfected with non specific or targeted against PR-B, Beclin-1 or Bcl-2 siRNA treated with vehicle (−) or 10 nM OHPg for 96h. (magnification = 400×). Images are representative of three independent experiments. DAPI staining not shown **B.** Percentage of positive SAβGal cells was determined from quantitating three fields at 200× magnification. Values were normalized to total nuclei present in each field from DAPI staining *p ≤ 0.05 vs vehicle; ** p ≤0.05 vs NS siRNA 10 nM OHPg **C.** Western blot analysis of pRb, p53 and p21 expression in T47-D and MCF-7 cells treated with 10 nM OHPg as indicated. β actin was used as loading control. Autoradiographs show the results of one representative experiment out of three. **D.** Real-time PCR assay of p16 and p14 mRNA expression. T47-D cells were treated with vehicle (−) or 10 nM OHPg as indicated. 18S rRNA was determined as control. Columns are the mean of three independent experiments each in triplicate; bars, SD; * p ≤0.05 vs vehicle treated cells.

The capability of OHPg/PR-B to promote the senescence process was demonstrated by the senescence-associated β-galactosidase (SAβGal) staining [34]. T47-D and MCF-7 cells exposed to 10 nM OHPg for 96h evidenced 40-60% SAβGal positivity relative to minimal SAβGal (10-15%) in vehicle-treated cells (Figure 7A, 7B). Worthy of note, specific silencing of PR-B, Beclin-1 or Bcl-2 greatly decreased the OHPg induced SAβGal positivity. This supports the idea that OHPg *via* PR-B/Bcl-2 axis drives breast cancer cellular senescence which follows an early autophagy mediated by Beclin-1.

Senescence is accompanied by a number of characteristic changes in gene expression [35]. Among these, the modulation of the p53/p21Cip1/Waf1 (p21) and p14ARF or p16Ink4a (p16) appears to be functionally relevant for the establishment and maintenance of the senescent state.

Protein content for p21 and p53 were scarcely influenced by OHPg treatment (Figure 7C) while, as expected, a dephosphorylation of Rb was evidenced especially after 48h of treatment, which was sustained until 96h in T47-D cells. In line with this, we observed the up-regulation of p16 mRNA levels starting from 48h until 96h (Figure 7D), while p14ARF remained unaltered by OHPg treatment. Similar results were obtained in MCF-7 cells (data not shown). These data indicate that the p16-pRb signaling is influenced by OHPg, suggesting this could be a pathway implicated by OHPg-mediated cellular senescence.

## DISCUSSION

Autophagy is largely considered a catabolic process by which cellular components are recruited to lysosomes for their degradation. The insufficient remodeling of damaged proteins and organelles can led to the efficient transition to a senescent phenotype characterized by an irreversible growth arrest [31].

Actually there is an increasing evidence for autophagic inhibition of cancer cell survival by novel cancer drugs and the mechanisms involved in the different paradigms are very diverse and complex. A draft scenario of the key molecular targets involved is emerging and in this concern our recent acquisition underlines a functional interplay between OHPg/PR-B and PTEN stimulating autophagy initiation in breast cancer cells, further supporting a protective role of OHPg/PR-B in breast cancer [36]. Herein, we demonstrate for the first time that OHPg/PR-B induced autophagy drives to senescence in breast cancer cell models, as evidenced by several featuring markers.

We define the molecular mechanisms by which PR-B specific ligand OHPg determines autophagy initiation, through the up-regulation of the master regulator Beclin-1. OHPg action occurs at transcriptional level as demonstrated by functional studies. Interestingly, we identified the presence of a canonical 1/2PRE site, crucial for Beclin-1 promoter induction by OHPg. We evidenced that the binding of PR-B to the identified OHPg-responsive sequence favors the recruitment of the SRC-2 coactivator and RNA Pol II to the promoter.

Beclin-1 induces autophagy by participating both in the biogenesis and degradation of autophagosomes *via* its interaction with different molecular complexes. Among Beclin-1-binding proteins, Bcl-2 is an important inhibitor for autophagy [31, 33]. The dissociation of Beclin-1 from Bcl-2 is regulated by many proteins and signal pathways and is necessary for autophagy induction.

An important finding in this paper is the significant increase of the functional Beclin-1 amount, resulting from the cooperation of two separate mechanisms stimulated by OHPg/PR-B: a) the increase of Beclin-1 expression b) the release of Beclin-1 from the inhibitory Bcl-2 interaction. The latter event is due to Bcl-2 phosphorylation, crucially determined by rapid and sustained JNK activation, thus enhancing the extent of Beclin-1 useful for autophagosomes assembly.

From our studies it emerges that the functional release of Beclin-1 from Bcl-2 however did not produce the efficient autophagic flux. Indeed p62 levels were not substantially reduced after OHPg stimulus [29, 37, 38]. We retain that the impaired autophagy could facilitate the irreversible growth arrest of breast cancer cells, induced by Bcl-2 *via* p27. Thus, the functional release of Bcl-2 from Beclin-1 interaction may represent the molecular bridge connecting autophagy to an irreversible G1 arrest, thus determining breast cancer cell fate decision.

For instance together with its well-established function in governing cell survival [39], Bcl-2 has been found to influence the cell cycle [32, 40]. Elevated Bcl-2 levels are associated with decreased proliferation and sometimes also a favorable prognosis in diverse malignancies, including breast [41]. Bcl-2 can both accelerate withdrawal from the cycle [42] and retard reentry [43] by increasing p27 [33] which inhibits G1-S progression [44]. Our study shows that OHPg/PR-B *via* Bcl-2 determine the induction of p27 and the cellular senescent phenotype. It is worth to mention that the specific silencing of Bcl-2 and PR-B reverse the effects produced by OHPg on p27 expression as well as the staining for SAβGal activity, marker of cellular senescence.

**Figure 8:**
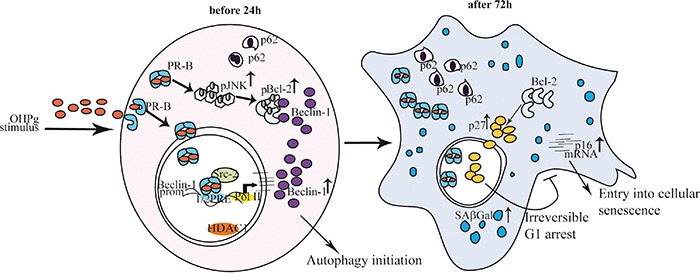
Proposed model for OHPg/PR-B-induced autophagy-senescence transition in ER+ breast cancer cells See text for details.

Thus, the most convincing interpretation of our results is that OHPg, by enhancing the levels of functional Beclin-1, causes the establishment of an early but incomplete autophagic process, linked to the release of Bcl-2 causing the induction of p27, which in turn leads to the irreversible inhibition of cell cycle progression (Figure 8). Indeed Beclin-1 depletion, which greatly attenuated the activation of autophagy by OHP as evidenced by TEM, also counteracted the establishment of senescence features. In other words the present paper highlights how OHPg triggering autophagy as early event, may create the susceptibility to cellular senescence.

In summary, we have demonstrated a significant role for OHPg/PR-B for Beclin-1 induction and function beginning from the autophagy initiation in breast cancer cells. Our findings thus indicate that an important tumor-suppressive pathway through Beclin-1 is target of OHPg *via* its own receptor, actually proposing a distinctive protecting action against breast cancer. Future studies may lead to additional strategies for the current therapy of breast cancer patients.

## MATERIALS AND METHODS

### Reagents

17-Hydroxyprogesterone (OHPg), aprotinin, leupeptin, phenylmethylsulfonyl fluoride (PMFS), sodium orthovanadate, NaCl, MgCl2, EGTA, glycerol, Triton X-100, Fetal Calf Serum (FCS), Fetal bovine serum (FBS), HEPES were from Sigma-Aldrich (Milan-Italy). Antibodies against human PR, β-actin, p27, Bcl-2, p21, p53, RNA Pol II, SRC-2, p-JNK, p-MAPK (ERK1, ERK2), JNK, MAPKs (ERK1, ERK2) and Protein A/G PLUS-Agarose were from Santa Cruz Biotechnology (Santa Cruz, CA). Antibodies to p-p38, p-38, p62, p-pRb and Beclin-1 were from Cell Signaling (Beverly, MA). RU 486, SB203580, PD98059, SP600125 were from Calbiochem.

### Plasmids

The firefly luciferase reporter plasmid containing the full-length of the Beclin1 promoter region [p-644 (−644/+197)] and the different deletion constructs [p-277 (−277/+197), p-58 (−58/+197)] gifts from Dr. Mujun Zhao (Institute of Biochem & Cell Biology, Chinese Academy of Sciences, China) [21]. The full-length PR-B consisting of the full-length PR-B cDNA fused with the SV40 early promoter and expressed in the pSG5 vector gift from Dr. D. Picard, (University of Geneva, Switzerland); the full length PR-A provided by Prof. Paul Kastener (Laboratary of Moleculare Genetic, CNRS, Strasbourg, France) [45]. PR DNA-binding mutant C587A (mDBD PR) was provided by Dr. C. Lange (University of Minnesota Cancer Center, Minneapolis, MN) [46] The Renilla luciferase expression vector pRL-TK (Promega) was used as a transfection standard.

### Cell culture

T47-D, MCF-7 and MDA-MB-231 human breast cancer cells were obtained from the American Type Culture Collection (ATCC). MCF-7 were maintained in DMEM/F-12 medium containing 5% FCS, 1% L-glutamine, 1% Eagle's nonessential amino acids, and 1 mg/ml penicillin/streptomycin in a 5% CO2 humidified atmosphere. T47-D cells were routinely maintained in RPMI 1640 supplemented with 5% FCS, 1μg/ml insulin (Sigma, Milan, Italy), 1 mg/ml penicillin/streptomycin (Sigma, Milan, Italy). MDA-MB-231 were maintained in DMEM/F-12 medium containing 5% FBS. Hormone stimulation was performed in medium containing 5% charcoal-treated FCS to reduce the endogenous steroid concentration [47].

### Transmission electron microscopy (TEM)

Transmission Electron Microscopy was conducted as previously described [9]. Briefly, cells treated as indicated were fixed in 3% glutaraldehyde (Sigma-Aldrich, Milan-Italy) solution in 0.1 M phosphate buffer (pH. 7.4) for 2h. Then the samples were post-fixed in osmium tetroxide (3%), dehydrated in graded acetone, and embedded in Araldite (Sigma-Aldrich, Milan-Italy). Ultrathin sections were collected on copper grids and contrasted using both lead citrate and uranyl acetate. The grids were examined in a “Zeiss EM 10” electron microscope.

### Total RNA extraction, reverse transcription polymerase PCR and real-time RT-PCR assay

Total RNA was extracted from T47D and MCF-7 cells using TRIzol reagent and cDNA was synthesized by reverse transcription-polymerase chain reaction (PCR) method using a RETROscript kit. The expression of selected genes was quantified by real-time PCR using iCycler iQ Detection System (Bio-Rad, Hercules, CA).

Five microliters of diluted (1:4) cDNA was analyzed using SYBR Green Universal PCR Master Mix, following the manufacturer's recommendations. The primers all from Invitrogen were: 5′-CCTGGAGGCGGCGAGAAC-3′ (p14 forward), 5′-CAGCACGAGGGCCACAGC-3′ (p14 reverse), 5′-CTTGCCTGGAAAGATACCG-3′ (p16 forward), 5′-CCCTCCTCTTTCTTCCTCC-3′ (p16 reverse),. (Beclin-1 forward) 5′-AGCTGCCGTTATACTGTTCTG-3′; (Beclin-1 reverse) 5′-ACTGCCTCCTGTGTCTTCAATCTT-3′ [48]. PCRs were performed in the iCycler iQ Detection System (Bio-Rad), using 0.1μM each primer in a total volume of 30 μL of reaction mixture following the manufacturer's recommendations. Each sample was normalized on the basis of its 18S ribosomal RNA content. The 18S quantification was performed using a TaqMan Ribosomal RNA Reagent kit (Applied Biosystems) following the method provided in the TaqMan Ribosomal RNA Control Reagent kit. The relative gene expression levels were normalized to a calibrator that was chosen to be the basal, untreated sample. Final results were expressed as n-fold differences in gene expression relative to 18S ribosomal RNA and calibrator, calculated following the ΔΔThreshold cycle (Ct) method, as published previously [49]. Assays were performed in triplicate.

### Western blotting and immunoprecipitation

Cells were grown in 10 cm dishes exposed to treatments for different times as indicated and lysed. Total extracts were prepared and subjected to SDS-PAGE as previously described [50].

For Immunoprecipitation, 500 μg of protein lysates were incubated overnight with the specific antibody and 500 μL of HNTG buffer [50 mmol/L HEPES (pH 7.4), 50 mmol/L NaCl, 0.1% Triton X-100, 10% glycerol, 1 mmol/L phenylmethylsulfonyl fluoride, 10 Ag/mL leupeptin, 10 Ag/mL aprotinin]. Immunocomplexes were recovered by incubation with protein A/G-agarose. The beads containing bound proteins were washed by centrifugation in immunoprecipitation buffer, then denatured by boiling in Laemmli sample buffer and analyzed by Western blot. The images were acquired by using an Epson Perfection scanner (Epson, Japan) using Photoshop software (Adobe). The optical densities of the spots were analyzed by using ImageJ software (NIH; http://rsb.info.nih.gov/IJ).

### Transfections and luciferase assays

Transfections were done as described [51] using Fugene 6 reagent (Roche Diagnostics, Milan, Italy). Luciferase activity was measured with the Dual Luciferase kit (Promega, Milan, Italy).

### Site-directed mutagenesis

Mutagenesis was performed on pGL3-644 of the Beclin-1 promoter by PCR. The sequence for the sense primer was:

5′-ATATAGATCTCCTTTTGTTGTTGCTGTTGT*TACCCT*GAGATGGAGCCTTGCCCTGT-3′. The amplified DNA fragment was digested and ligated into pGL3-basic vector. Mutation was confirmed by DNA sequencing.

### Lipid-mediated transfection of siRNA duplexes

Cells were transfected with 4 functionally verified siRNA directed against human PR-B, Beclin1 or Bcl-2 or with a control siRNA (Qiagen, Mi, Italy) that does not match with any human mRNA used as a control for non-sequence specific effects. Cells were transfected using Lipofectamine 2000 reagent (Invitrogen, Paisley, UK) and then treated as indicated.

### Chromatin immunoprecipitation (ChIP) assays and realtime ChIP

Cells were treated as indicated before harvesting for the assay performed as described [52].

The immuno-cleared chromatin was precipitated with antibodies as indicated. Immunoprecipitated DNA was analyzed in triplicates by real-time PCR by using 5 μl of the diluted (1:3) template DNA or PCR. The following primers, corresponding to the region between −629 bp to −514 bp, used for PCR: forward 5′ ATTTTGGCCAGGCTGGTCT 3′ and reverse 5′ GCACGCCTATAATCCCAGCT 3′ were used (Invitrogen, Paisley, UK). Real-time PCR data were normalized with respect to unprocessed lysates (input DNA). Inputs DNA quantification was performed by using 5 μl of the diluted (1/50) template DNA. The relative antibody bound fractions were normalized to a calibrator that was chosen to be the basal, untreated sample. Final results were expressed as percent to the relative inputs as previously described [53].

### Flow cytometry

T47-D and MCF-7 cells, treated with OHPg 10 nM for different times as indicated were collected for cell cycle analysis. Cells were washed with PBS and fixed for 1h in ice-cold 70% ethanol. The samples were then washed once with PBS and suspended in 1ml of staining solution (10 mg/ml RNasi A, 10 mg/ml propidium iodide in PBS) and then incubated at room temperature in the dark for at least 30 min. FACS analysis was performed using ModFit LT software (Becton Dickinson, NJ). At least 2×10^4^ cells/sample were measured.

### Senescence associated-β-galactosidase (SAβGal) activity assays

T47-D and MCF-7 cells were continuously treated in the presence or absence of 10 nM OHPg for 96h. Cells were washed, fixed and stained for SAβGal activity according to manufacturer's instructions using the Senescence β-Galactosidase Staining Kit (Cell Signaling Technology, #9860).

### Statistical analysis

Data were analyzed by Student's t test using the GraphPad Prism 4 software program. and results were presented as mean ± SD. A value of P≤ 0.05 was considered to be significant.

## SUPPLEMENTARY FIGURE


