# Supplementary material for: Ligand activated progesterone receptor B drives autophagy-senescence transition through a Beclin-1/Bcl-2 dependent mechanism in human breast cancer cells
Source: Oncotarget. 2016 Jul 23;7(36):57955–69. doi: 10.18632/oncotarget.10799 (PMC5295403; doi:10.18632/oncotarget.10799)
Supplement: Supplementary file 1 [file oncotarget-07-57955-s001.pdf]

Ligand activated progesterone receptor B drives autophagy-senescence transition through a Beclin-1/Bcl-2 dependent mechanism in human breast cancer cells

SUPPLEMENTARY FIGURE

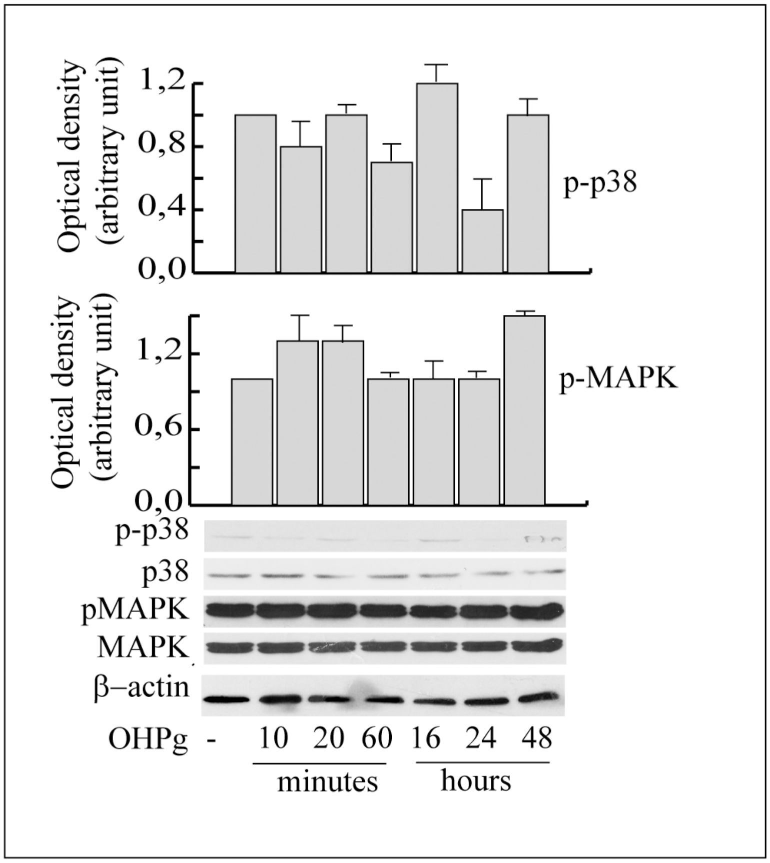

Supplementary Figure S1: Western blotting analysis of p-p38/p-38 and pMAPK/MAPK. T47-D cells were treated with 10 nM OHPg as indicated. beta-actin was used as loading control. Autoradiographs show the results of one representative experiment. Columns, are mean of three independent experiments in which band intensities were evaluated in terms of optical density arbitrary units and expressed as fold over vehicle, which was assumed to be 1; bars, SD.
